# Comprehensive Analyses of Advanced Glycation end Products and Heterocyclic Amines in Peanuts during the Roasting Process

**DOI:** 10.3390/molecules28207012

**Published:** 2023-10-10

**Authors:** Jingjing Yu, Xiaohui Yu, Lili Shi, Wei Liu

**Affiliations:** 1 Zhengzhou Tobacco Research Institute of CNTC, Zhengzhou 450001, China; 2 College of Food Science and Technology, Henan University of Technology, Lianhua Street, Zhengzhou 450001, China; xiaohuiyu0112@126.com (X.Y.); shililislla@163.com (L.S.)

**Keywords:** advanced glycation end products, heterocyclic amines, peanut, roasting, correlation analysis

## Abstract

Advanced glycation end products (AGEs) and heterocyclic amines (HAs) are two kinds of important harmful products formed simultaneously during the thermal processing of proteinaceous food. In this paper, the effect of roasting conditions on the formation of AGEs and HAs, as well as active carbonyl intermediates in common peanut (C−peanut) and high-oleic acid peanut (HO−peanut) was studied simultaneously for the first time. In general, with the increase in roasting temperature (160–200 °C) and time, the contents of AGEs, HAs and active carbonyl intermediates (i.e., glyoxal (GO) and methylglyoxal (MGO)) significantly increased in peanuts. Four kinds of HAs (i.e., AαC, DMIP, Harman and Norharman) were observed in roasted peanuts, of which Harman and Norharman accounted for about 93.0% of the total HAs content after roasting for 30 min at 200 °C. Furthermore, a correlation analysis among AGEs (i.e., *N*^ε^-(1-Carboxymethyl)-L-lysine (CML) and *N*^ε^-(1-Carboxyethyl)-L-lysine (CEL)), HAs, GO and MGO was conducted. Most of these compounds showed an excellent positive linear relationship (*p* ≤ 0.001) with each other. The evident increase in GO and MGO contents implied an increase in not only the content of AGEs but also HAs. However, contents of AGEs and HAs showed no significant difference between roasted HO−peanut and C−peanut. This study would provide a theoretical basis for simultaneously controlling the levels of AGEs and HAs in thermal processed peanut foods.

## 1. Introduction

Peanut (*Arachis hypogaea* L.) is one of the major global oilseeds and a widely consumed food in most countries, such as China, the USA and many European countries. Peanut contains fats (45–55%), proteins (22–30%), carbohydrates (15–21%) and other natural substances (e.g., vitamins). The fats of peanut is mostly composed of oleic acid (monounsaturated fatty acid, C18:1) and linoleic acid (polyunsaturated fatty acid, C18:2), which are essential fatty acids responsible for some health-promoting effects. And peanut is an important source of high-quality protein, which contains all essential amino acids necessary for normal human growth and metabolism [1]. Moreover, peanut contains some minor components such as phenolics, flavonoids, tocopherols and squalene, which provide antioxidant, anti-inflammatory and anti-cancer properties [2]. One of the most of consumed forms of peanut is roasted peanut, which results in the development of flavor. As one of the main thermal processing methods, roasting can result in favorable flavor and color, and change nutritional properties and allergenicity of peanuts, and it is conducted at high temperature range of 150–200 °C [3,4,5,6,7,8,9]. During the thermal processing, many food chemical reactions take place, such as the Maillard reaction, lipid oxidation, Strecker condensation, and so on.

On the other hand, thermal food processing (e.g., roasting) can generate some undesirable risk factors, such as advanced glycation end products (AGEs) and heterocyclic amines (HAs) [10,11,12,13]. AGEs are harmful chemicals formed in non-enzymatic reactions, which are related to the polymerization, condensation, and other reactions between the free amino groups of proteins and the active carbonyls of reducing sugars or intermediate carbonyl compounds of the Maillard reaction. Endogenous AGEs from the daily diet can accumulate in vivo and cause many harmful effects, such as increased oxidative stress, inflammation, and other chronic diseases [14,15]. *N*^ε^-(1-Carboxymethyl)-L-lysine (CML) and *N*^ε^-(1-Carboxyethyl)-L-lysine (CEL) are considered as two typical markers of AGEs, which are usually used to evaluate the AGEs level [16]. As for HAs, they are a class of mutagenic and carcinogenic compounds formed through the Maillard reaction when creatinine, carbohydrates and amino acids are heated at higher temperatures (150–250 °C) [17]. Based on the results of epidemiological and animal studies, most HAs are classified by IARC as Class 2A and 2B human carcinogens [17,18]. Active carbonyl intermediates such as glyoxal (GO) and methylglyoxal (MGO) [19] are markers of thermal processing. Generally, the formation of active carbonyl intermediates, AGEs and HAs, occurs simultaneously in foods during thermal processing. However, most of AGEs and HAs in thermal processing food have been studied separately [20,21,22,23,24], which cannot provide a full insight for controlling the level of food risk factors. There are only a few examples. Quan et al. have revealed a report focusing on the simultaneous generation of acrylamide, carboline HAs and AGEs in an aqueous Maillard reaction model system [25]. Xue et al. have investigated the formation of HAs and AGEs in roast beef patties [26].

Thermal food processing can lead to the formation of undesirable risk factors, such as AGEs and HAs. Thus, it is important to determine food risk factors simultaneously, which would provide more insights into the correlations between different food risk factors (e.g., AGEs and HAs). Since AGEs and HAs are important harmful products formed simultaneously during the thermal processing of proteinaceous food, the influence of roasting conditions (i.e., roasting temperature and time) on the contents of AGEs and HAs, as well as GO and MGO in roasted peanuts were studied in this paper. Furthermore, a correlation analysis among AGEs (CML and CEL), HAs and the active intermediates (GO and MGO) were conducted. This study will provide a theoretical basis for simultaneously controlling the level of AGEs and HAs in peanut thermal food processing.

## 2. Results and Discussion

### 2.1. Method Validations of AGEs, HAs, GO and MGO

Method validations of AGEs, HAs, GO and MGO were conducted, and the results are shown in Table 1. As CML and CEL are two typical markers of AGEs, limit of detections (LODs) and limits of quantification (LOQs) of CML and CEL analysis were evaluated in this study, and LODs were 0.1050 mg/kg and 0.0875 mg/kg, respectively. The average recoveries of CML and CEL in samples were 104.00% and 115.00%, respectively. Based on the sample analysis data, only four kinds of HAs (i.e., AαC, DMIP, Harman and Norharman) could be detected in roasted peanuts. Thus, method validations for four HAs were also conducted in this study. As shown in Table 1, LODs of four HAs were less than 0.0009 mg/kg, while their average recoveries were in the range of 81.91–91.17%. Furthermore, the intra-day precisions for both AGEs and HAs were excellent (RSD ≤ 9.43%). The developed GO and MGO method also showed good analytical performance, as shown in Table 1. The LODs of GO and MGO were 0.0160, 0.0180 mg/kg, respectively. These methods were suitable to determine the contents of AGEs, HAs, GO and MGO in peanuts in this study. 

### 2.2. Determination of AGEs and HAs Contents in Roasted Peanuts

In this paper, the contents of AGEs and HAs in peanuts before and after roasting (180 °C, 30 min) for different kinds of peanuts were investigated, as shown in Table 2. The contents of AGEs and HAs increased significantly after being roasted. The contents of CML in 10 kinds of peanuts without roasting were very lower (0–8.02 mg/kg), while CEL was undetected. The contents of CML and CEL in the roasted peanuts significantly increased with the contents in the range of 302.98–540.47 mg/kg and 81.38–114.63 mg/kg, respectively. It indicated that a violent Maillard reaction occurred during the roasting process of peanuts. In addition, the contents of CML were higher than CEL both in raw and roasted peanut samples. And the increase in CML in peanuts after being roasted was much higher than that of CEL, as shown in Table 2. However, no HAs were detected in the 10 kinds of raw peanuts, and the contents of HAs in peanuts increased after being roasted at 180 °C for 30 min (Table 2). As we know, the formation of HAs required higher temperature, generally above 150 °C [10,27], so HAs could not be detected in the raw peanuts. After being roasted at 180 °C for 30 min, only three kinds of HAs (i.e., DMIP, Harman and Norharman) were detected in the peanuts, and the contents were in the range of 2.78–12.45 µg/kg for DMIP, 3.48–16.20 µg/kg for Harman and 4.03–15.23 µg/kg for Norharman. It should be noted that AαC only could be detected when peanuts were roasted at 200 °C for 30 min. However, there were no significant differences in the contents of AGEs and HAs between C−peanuts (sample 1~7) and HO−peanuts (sample 8~10).

### 2.3. Effect of Roasting Conditions on GO and MGO Contents

**Figure 1:**
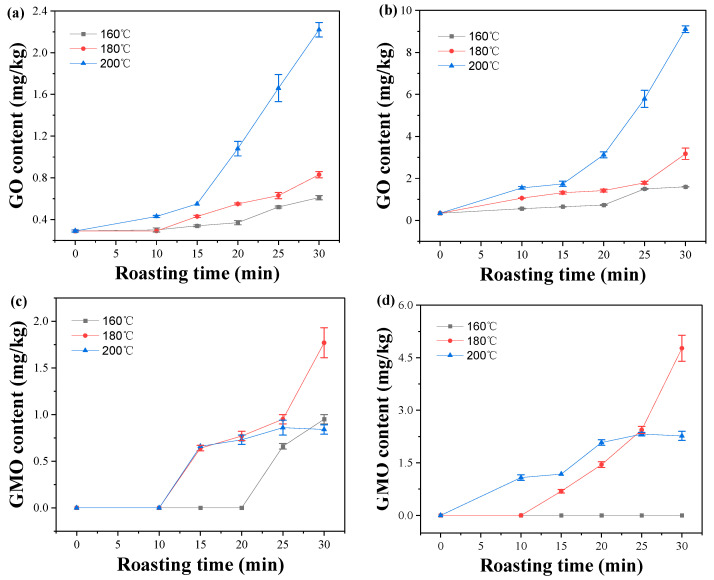
Effect of roasting conditions on GO contents in C−peanut (**a**) and HO−peanut (**b**), and MGO contents in C−peanut (**c**) and HO−peanut (**d**).

GO and MGO are important intermediate substances for the formation of CML and CEL. Furthermore, they can also participate in the Strecker degradation reaction, thus indirectly participating in the formation of HAs [28]. Both a typical C−peanut sample (sample 2) and a typical HO−peanut sample (sample 10) were selected to study the effect of different roasting conditions on GO and MGO contents in this study. The content of oleic acids in C−peanut and HO−peanut was 43.56% and 79.11%, respectively. As shown in Figure 1, the contents of GO in two kinds of raw peanuts (C−peanut, HO−peanut) were very low, and MGO was not detected. The contents of GO significantly increased with the increase in roasting time and temperature. And the contents of GO in roasted peanuts were similar at 160 °C and 180 °C. However, when the roasting temperature was 200 °C and the roasting time was more than 15 min, the contents of GO in the two kinds of peanuts were significantly much higher than that at 160 °C and 180 °C. It indicated that higher roasting temperatures led to higher contents of the GO. As for MGO, it could not be detected at the first 20 min at 160 °C in C−peanut, but it rapidly increased when roasting was conducted over 20 min. However, MGO could not be detected in HO−peanut after roasting at 160 °C. It indicated that MGO was difficult to form below 160 °C in the two kinds of peanuts. At 180 °C, the contents of MGO in peanuts rapidly increased with roasting time. At 200 °C, the MGO contents in C−peanut and HO−peanut increased with time at the preceding stage but were lower than those for roasting at 180 °C for over 20 min. It was related that the Maillard reaction at a higher temperature would consume large amount of MGO. And the formation of AGEs and HAs also take place rapidly at 180–200 °C [29,30]. As for C−peanut, the fatty acid composition of peanut oil contained saturated fatty acids (e.g., C16:0, C18:0) 22.73%, mono-unsaturated fatty acids (e.g., C18:1) 44.32% and poly-unsaturated fatty acids (e.g., C18:2) 32.95%. As for HO−peanut, the fatty acid composition of peanut oil contained saturated fatty acids (e.g., C16:0, C18:0) 16.92%, mono-unsaturated fatty acids (e.g., C18:1) 80.22% and poly-unsaturated fatty acids (e.g., C18:2) 2.86%. In general, HO−peanut oil is more stable than C−peanut oil. However, higher content levels of GO and MGO in HO−peanut was observed in this study. As we know, lipids oxidation/degradation and carbohydrates degradation all play important roles in the formation of 1,2-dicarbonyl compounds (e.g., GO and MGO) in food [31,32]. Moreover, the contents of reducing sugars in the peanuts during roasting are detected (Table S1). As for C−peanut at 200 °C, the contents of reducing sugars decreased from 2.23 g/kg (0 min) to 1.84 g/kg (10–20 min) and then gradually increased to 2.28 g/kg (30 min). As for HO−peanut at 200 °C, the contents of reducing sugars decreased from 2.44 g/kg (0 min) to 2.25 g/kg (20 min) and then rapidly increased to 3.38 g/kg (30 min). Based on the contents of reducing sugars (CRS) in different peanuts, it was found that the change in CRS in peanuts occurred in the following order: HO−peanut > C−peanut (Figure S1). Thus, the content levels of GO and MGO in different peanut samples after roasting was in line with the change in CRS in peanuts. 

### 2.4. Effect of Roasting Conditions on CML and CEL Contents in Peanuts

**Figure 2:**
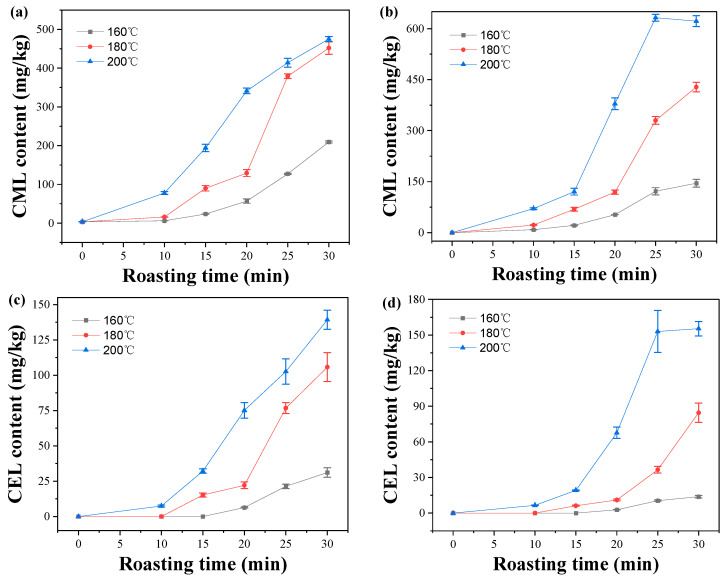
Effect of roasting conditions on CML contents in C−peanut (**a**) and HO−peanut (**b**), and CEL contents in C−peanut (**c**) and HO−peanut (**d**).

Roasting temperature and time are very important factors affecting the formation of AGEs (e.g., CML and CEL) in food. In this study, the effect of roasting conditions on the contents of CML and CEL in C−peanut (sample 2) and HO−peanut (sample 10) were investigated, and the results are shown in Figure 2. It can be seen that with the increase in roasting temperature and time, the contents of CML and CEL in C−peanut and HO−peanut significantly increased. However, at 200 °C, the contents of CML and CEL in HO−peanut did not increase when roasted over 25 min. And there were linear relationships between CML or CEL content and roasting time, except for zero point, while all of the correlation coefficients were greater than 0.8792 at 160 °C, 180 °C, and 200 °C. It could be concluded that higher slope of fitting straight line indicated a faster formation rate. Thus, the higher roasting temperature led to the faster formation rate of CML and CEL. Furthermore, the formation rate of CML was faster than that of CEL, which was related to the higher activation energies for the formation of CEL than that of CML [13]. In addition, the formation rates of CML and CEL in HO−peanut were slightly lower than those in C−peanut at 160 °C and 180 °C, while the formation rates in HO−peanut were slightly higher than those in C−peanut at 200 °C.

### 2.5. Effect of Roasting Conditions on HAs Contents in Peanuts

The contents of HAs in C−peanut and HO−peanut under different roasting conditions are shown in Table 3 and Table 4, respectively. As seen, with the increase in roasting temperature and time, the varieties and contents of HAs in the peanuts increased. It could be observed that under the roasting temperature of 160–200 °C, four kinds of HAs, including 2-amino-9H-pyrido[2,3-b] indole (AαC), 2-amino-1,6-dimethylimidazo[4,5-b] pyridine (DMIP), 1-methyl-9H-pyrido[3,4-b] indole (Harman), 9H-pyrido[3,4-b]indole (Norharman) were detected in both C−peanut and HO−peanut, while only three HAs (DMIP, Harman and Norharman) could be detected at 160–180 °C. AαC was detected in both two peanuts at 200 °C for 30 min, indicating that proteinous foods (e.g., peanut) could also produce such “thermic HAs” when being heated for enough time below 250 °C (such as 200 °C) [27]. AαC is a non-polar HAs, which is usually produced by the pyrolysis of amino acids or proteins at high temperatures above 250 °C [33]. 

In addition, with the increase in roasting temperature, the content of DMIP in all the peanuts significantly increased, and though the content of DMIP in C−peanut was 0.82 ± 0.07 µg/kg, it was not detected in HO−peanut at 160 °C for 30 min (Table 4). Harman and Norharman were the two major HAs in roasted peanuts, and their contents significantly increased with the increase in roasting temperature and roasting time [27]. Especially, for roasting at 200 °C, their contents showed a sharp growth. When roasted at 200 °C for 30 min, Harman and Norharman contents of C−peanut accounted for 92.7% of the total HAs contents, while those in HO−peanut accounted for 92.6% of the total HAs contents. Notably, increasing the roasting temperature could significantly promote the formation of HAs in peanuts. Indeed, roasting temperature is especially important for the formation of HAs in food [29].

### 2.6. Correlation Analysis

**Figure 3:**
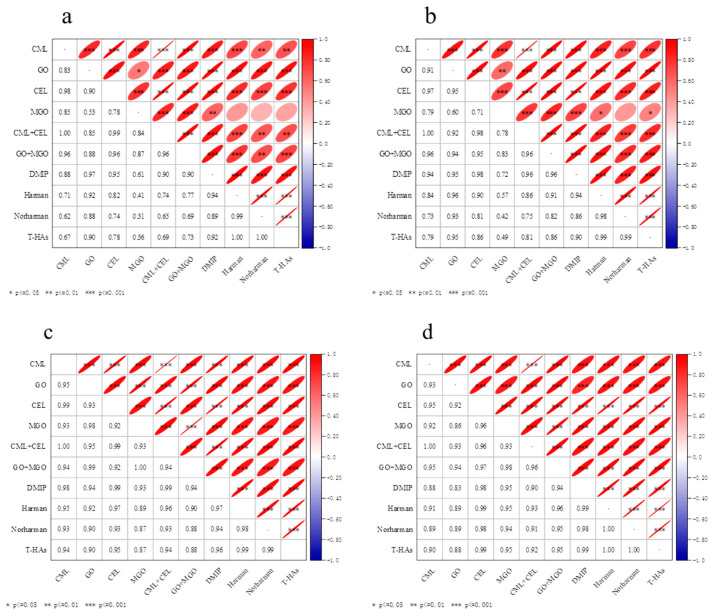
Correlation analysis of different compounds for C−peanut (**a**) and HO−peanut (**b**) after roasting at 160 °C, 180 °C and 200 °C; for C−peanut (**c**) and HO−peanut (**d**) at 160 °C and 180 °C. * *p* ≤ 0.05, ** *p* ≤ 0.01, *** *p* ≤ 0.001.

AGEs and HAs are the two kinds of risk factors during thermal food processing, while GO and MGO are the important intermediate substances of the Maillard reaction. Therefore, carrying out a correlation analysis among them is very important for food safety and processing. The correlation analysis among the contents of CML, GO, CEL, MGO, CML + CEL (i.e., total contents of CML and CEL), GO + MGO (i.e., total contents of GO and MGO), DMIP, Harman, Norharman and total HAs (T−HAs, i.e., total contents of AαC, DMIP, Harman and Norharman) in roasted peanuts were conducted in this study. Origin 2021 was used for data charting. As shown in Figure 3a,b, with the increase in the roasting temperature from 160 °C to 200 °C, most of the parameters showed a positive linear relationship (*p* ≤ 0.05), except those for Harman vs. MGO, Norharman vs. MGO, and T−HAs vs. MGO in C−peanut, as well as Norharman vs. MGO in HO−peanut. However, as shown in Figure 3c,d, with the increase in roasting temperature from 160 °C to 180 °C, all parameters showed an excellent positive linear relationship (*p* ≤ 0.001). The slightly poor correlation observed at 200 °C was due to the fast degradation reactions of these compounds that occurred at higher temperature [34,35], such as AGEs, GO and MGO, which was discussed above.

GO and MGO are the important intermediate substances for the formation of CML and CEL, respectively. The relationship between CML and CEL and their intermediate products GO/MGO was also discussed. At 160 °C and 180 °C, the contents of CML vs. GO, and CEL vs. MGO in C−peanut showed a linear relationship with *r* of 0.95 and 0.92 (*p* ≤ 0.001), respectively. Furthermore, the contents of CML vs. GO, and CEL vs. MGO in HO−peanut showed a linear relationship with *r* of 0.93 and 0.96 (*p* ≤ 0.001), respectively. At all three roasting temperatures (160 °C, 180 °C, and 200 °C), the total contents of CML and CEL (CML + CEL) showed a good linear relationship with the total content of GO and MGO (GO + MGO) with *r* of 0.96 (*p* ≤ 0.001) in two kinds of roasting peanuts. It indicated that GO/MGO was an important indicator for AGEs. Furthermore, at 160 °C and 180 °C, as for two typical and important AGEs, the contents of CML and CEL both in C−peanut and HO−peanut showed a good linear relationship with their correlation coefficients (*r*) of 0.98 and 0.97, respectively. It indicated that the formation mechanism of CML and CEL was similar in a food system. Furthermore, the levels of CML and CEL showed a linear relationship with the total content of CML and CEL as well.

As for the HAs, three kinds of HAs were mainly detected in the roasted peanuts. The content of DMIP, Harman, Norharman and total HAs (T−HAs) showed an excellent positive linear relationship (*p* ≤ 0.001) with roasting temperature at 160–200 °C. As GO and MGO are the typical intermediate substances of the Maillard reaction, the relationships between them and HAs were also discussed. At 160 °C and 180 °C, for C−peanut and OH−peanut, *r* for DMIP, Harman, Norharman, T−HAs vs. GO were above 0.83 (*p* ≤ 0.001), while *r* for those vs. MGO were above 0.87 (*p* ≤ 0.001), and *r* for those vs. the total content of GO and MGO (GO + MGO) were above 0.90 (*p* ≤ 0.001).The evident increase in the GO and MGO contents implied an increase in both the harmful compounds, AGEs and HAs. 

As the two kinds of harmful compounds produced during thermal food processing, the relationships between AGEs and HAs was also discussed. At 160 °C and 180 °C, for C−peanut and OH−peanut, *r* for CML, CEL, CML + CEL *vs.* DMIP were above 0.88 (*p* ≤ 0.001), while *r* for them vs. Harman were above 0.91 (*p* ≤ 0.001), while *r* for them vs. Norharman were above 0.89 (*p* ≤ 0.001), while *r* for them vs. T−HAs were above 0.90 (*p* ≤ 0.001). It indicated that the contents of AGEs showed a linear relationship with the contents of HAs in the food system.

## 3. Materials and Methods

### 3.1. Materials and Reagents

Ten different varieties of peanuts were obtained from major peanut-producing provinces of China. There were 7 kinds of common peanuts (C−peanut), whose sample numbers were 1~7 (sample 1, Jihua 2, from Shandong Province, China; sample 2, Xiaobaisha, from Henan Province, China; sample 3, Yuhua, from Henan Province, China; sample 4, Baisha 308, from Henan Province, China; sample 5, Silihong 308, from Henan Province, China; sample 6, Tianfu 3, from Henan Province, China; sample 7, Luhua 8, from Shandong Province, China). There were 3 kinds of high oleic acid peanuts (HO−peanut), whose sample numbers were 8~10 (sample 8, Yuhua 65, from Henan Province, China; sample 9, Yuyan, from Henan Province, China; sample 10, Jihua 16, from Shandong Province, China). And the contents of oleic acid in the oil from peanut samples 1~10 were 41.42%, 43.56%, 36.25%, 41.07%, 44.38%, 42.02%, 41.52%, 75.92%, 76.38% and 79.11%, respectively.

The standards of CML, CEL, *N*^ε^-(1-Carboxymethyl)-L-lysine-*d*_4_ (CML-*d*_4_) and *N*^ε^-(1-Carboxyethyl)-L-lysine (CEL-*d*_4_) were purchased from Toronto Research Chemicals (Canada), while CML-*d*_4_ and CEL-*d*_4_ were used as internal standards. The standards of 2-amino-9H-pyrido[2,3-b] indole (AαC), 2-amino-1,6-dimethylimidazo[4,5-b] pyridine (DMIP), 1-methyl-9H-pyrido[3,4-b] indole (Harman), 9H-pyrido[3,4-b]indole (Norharman) and 4,7,8-TriMeIQx were purchased from Alta scientific (Tianjin, China), while 4,7,8-TriMeIQx was used as an internal standard. GO (40% aqueous solution) and MGO (40% aqueous solution) were purchased from Sigma-Aldrich (Shanghai, China). 4-nitro-o-phenylenediamine (≥97%) was obtained from Shanghai Aladdin Biochemical Technology Co., Ltd. (Shanghai, China). Acetonitrile, methanol and n-hexane were of HPLC grade and purchased from Dikama Corp. (Shanghai, China). Acetic acid and formic acid were of HPLC grade and obtained from TEDIA Company Inc. (Fairfield, CT, USA). Hydrochloric acid, sodium hydroxide, boric acid and sodium borohydride were analytical reagents and obtained from Sinopharm Chemical Reagent Beijing Co., Ltd. (Shanghai, China). Ethyl acetate was of analytical reagent and obtained from Tianjin Kemio Chemical Reagent Co., Ltd. (Tianjin, China). Ammonium hydroxide was obtained from CNW Technologies (Shanghai, China). Water was purified with a Milli-Q50 system (Millipore, Bedford, MA, USA).

### 3.2. Preparation of Roasted Peanuts

The roasting method for peanuts was conducted with minor modification based on a previous report [9]. High-quality peanut kernels with uniform color, full particles, uniform size and no mildew were selected and weighed (100 g) and then roasted in the oven (Henan Taist Instrument Corp., Zhengzhou, China) preheated to the set temperature (160 °C, 180 °C and 200 °C, respectively). After being roasted for a certain time (10 min, 15 min, 20 min, 25 min and 30 min, respectively), the peanuts were taken out and cooled naturally at room temperature. Then, they were crushed into powder using a grinder (Yongguangming Instrument Corp., Beijing, China) and frozen in a vacuum freeze-dryer (Boyikang Instrument Corp., Beijing, China) for 24 h. Finally, the roasted peanut powder was put into a sealed bag and stored in a refrigerator (Haier refrigerator Corp., Qingdao, China) at −20 °C for analysis.

### 3.3. Determination of GO and MGO Contents in Peanuts

For the determination of GO and MGO contents in peanuts, the method reported by Cengiz et al. [19] was applied with minor modification. Briefly, 3.0 g freeze-dried samples were accurately weighed and placed in a 50 mL centrifuge tube. After adding 5 mL methanol, the samples were extracted with a homogenizer for 2 min. Then, the samples were frozen and centrifuged at 4 °C (8000 r/min, 10 min). Next, 1.0 mL supernatant was taken into a 30 mL pressure-resistant reaction tube, and 2.0 mL sodium acetate buffer (0.1 mol/L, pH = 3.0) and 1.0 mL 4-nitro-o-phenylenediamine were added into the mixture. Then, the derivative reaction was carried out for 20 min at 70 °C under stirring. The reaction was terminated by putting the tube in an ice water bath. After cooling, the sample was transferred to a 5 mL volumetric flask and the volume was fixed with methanol. After being filtered with a 0.45 µm cellulose acetate filter, the sample was analyzed with the high performance liquid chromatography-diode array detector (HPLC−DAD) method. Each sample was tested 3 times.

Sample analysis was conducted on an Agilent 1200 HPLC−DAD instrument (Agilent Technologies Technology Co., Ltd., Santa Clara, CA, USA). The chromatographic column was an Agilent ODS-3 column (250 × 4.6 mm, 5 μm), while other parameters were set as follows. Column temperature: 30 °C; flow rate: 1.0 mL/min; injection volume: 10.0 µL. The mobile phase was the mixture of methanol, water and acetonitrile with the ratio of 42:2:56 (*v:v:v*). The detection wavelength was 273 nm.

### 3.4. Determination of AGEs Contents in Peanuts

The determination of typical AGEs (i.e., CML and CEL) contents was according to Yu et al.’s method [24] with minor modification. Briefly, 40.0 mg freeze-dried sample was degreased with 6.0 mL n-hexane for 3 times. Next, the sample powder was dried under nitrogen, and then 1.5 mL 0.2 mol/L sodium borate solution (pH 9.2) and 1.0 mL 1.0 mol/L sodium borohydride was added and reduced at 4 °C overnight. Then, 2.6 mL concentrated HCl was added to react for 24 h under an oxygen-free environment in a sealed hydrolysis tube. Then, the mixture was filtered and diluted to 10.0 mL with distilled water. Next, 1.0 mL of the obtained solution was mixed with 1.0 mL distilled water and 100 µL internal standard (CML-*d*_4_ and CEL-*d*_4_) for the purification with a cation exchange solid phase extraction (SPE) column (ProElut PXC, 150 mg/6 mL, Dikma Corp., Shanghai, China), which was preconditioned with 2.0 mL methanol and 2.0 mL 0.1 mol/L HCl solution. After washing with 2.0 mL distilled water, the column was eluted with 5.0 mL 5% ammonia−methanol solution. The elution solution was dried with a nitrogen blowing concentrator (Caliper lifesciences Turbovap II, Boston, MA, USA), and then 1.0 mL distilled water was used to dissolve for HPLC−MS/MS analysis.

CML and CEL were determined using an Agilent 1200 HPLC instrument (Agilent Technologies Technology Co., Ltd., Santa Clara, CA, USA) coupled with an AB SCIEX Triple QUAD 4000 mass spectrometer (AB Sciex Instruments, Boston, MA, USA). An Agilent Proshell 120 SB C_18_ column (3.0 × 150 mm, 2.7 μm) was chosen for HPLC analysis with a flow rate of 0.3 mL/min, and the injection volume was 10.0 µL. The mobile phase A was water with 0.3% acetic acid, while B was acetonitrile with 0.3% acetic acid. The gradient elution program was as follows: 0–5.0 min, 100% A–80%A; 5.1–10.0 min, 80% A–10%A; 10.1–15 min, 100% A.

Mass detection conditions were as follows: ionization mode, positive ESI; ion spray voltage, 5000 V; ion source temperature, 550 °C; curtain gas, nitrogen; setting: 30 psi; ion source gas 1 (GS1), setting: 70 psi; ion source gas 2 (GS2), setting: 70 psi. Two ion pairs were chosen as qualitative ion pairs, and a quantitative ion pair was used with multiple reaction monitoring (MRM) mode. The parameters of MRM containing precursor ion, production ion, dwell time, collision energy (CE), declustering potential (DP) for CML, CEL and the internal standards are shown in Table 5.

### 3.5. Determination of HAs Content in Peanuts

The determination of HAs was conducted according to Liu et al.’s method [27] with minor modification. About 2.0 g sample was added into 10.0 mL acetonitrile-ethyl acetate (1:1, *v:v*) containing 1.0% acetic acid, with the addition of 10.0 μL 5.0 mg/L 4,7,8-TriMeIQx as the internal standard. The samples were fully mixed on a vortex oscillator for 3 min, followed by ultrasonic extraction for 10 min. Finally, it was frozen and centrifuged for 10 min (10,000 r/min). The extraction was repeated twice to collect all supernatant. The sample was purified with a cation exchange SPE column (ProElut PXC, 150 mg/6 mL, Dikma Corp., Shanghai, China), which was preconditioned with 5.0 mL of methanol and 5.0 mL of 0.1 mol/L hydrochloric acid/methanol (80:20, *v:v*). Then, the SPE column was washed with 5.0 mL water, 5.0 mL methanol and 5.0 mL methanol/ammonia/water (25:5:75, *v:v:v*), successively. Finally, 5.0 mL 5% ammonia–methanol solution was used for elution, and the eluent was dried with nitrogen. The mixture of 5% formic acid-acetonitrile (*v:v*) was added in constant volume to 10.0 mL and filtered through a 0.45 μm microporous filter for HPLC-MS/MS analysis.

A Shimadzu LC-20ADXR system (Shimadzu Corp., Kyoto, Japan) coupled with Triple Quad 3500 mass spectrometer (AB Sciex Instruments, Boston, MA, USA) was used to analyze HAs. Chromatographic separation was performed on an Agilent ZORBAX Eclipse XDB-C_18_ column (150 mm × 2.1 mm i.d, 3.5 μm) maintained at 35 °C. The gradient elution was achieved with a binary mobile phase of 5% formic acid/5 mM ammonium formate aqueous solution (A) and 5% formic acid/5 mM ammonium formate methanol solution (B) at a flow rate of 0.4 mL/min. The gradient elution program was as follows: 0–1.0 min, 5%B; 1.1–5.0 min, 60%B–80%B; 5.1–6.0 min, 80%B–95%B; 6.1–8.0 min, 95%B; 8.1–10.0 min, 5%B. The injection volume was 5.0 μL.

MS analysis was carried out with a positive ESI mode. MRM parameters were automatically optimized. The capillary voltage was 5.5 kV, and the ion source temperature was 550 °C. MRM parameters for HAs are summarized in Table 5.

### 3.6. Methodology Validation

Method characterization, including linear range, LOD and LOQ, was conducted. A series of CML and CEL working solutions were prepared with water using CML-*d*_4_ and CEL-*d*_4_ as internal standards. And a series of HAs working solutions were prepared with methanol using 4,7,8-TriMeIQx as an internal standard. LOD and LOQ of the methods were determined with 3 triples and 10 folds of signal-to-noise ratio (*S/N*). Recovery was studied with the spiking standard solution in 3 different concentration levels. And each concentration level was performed 3 times. And the same sample was analyzed on 5 times in one day to obtain the intra-day precision.

### 3.7. Statistical Analysis

All data derived from experiments performed in triplicate were expressed as mean ± standard deviation. Statistical analysis was performed with IBM SPSS 24.0 using one-way ANOVA, followed by Duncan’s post hoc test, and *p*-value of < 0.05 was regarded as statistically significant.

## 4. Conclusions

In this study, the effect of roasting on the simultaneous formation of active carbonyl intermediates, AGEs and HAs, in peanuts was investigated. Overall, with the increase in roasting temperature and time, the contents of GO, MGO, CML, CEL and four HAs (AαC, DMIP, Harman and Norharman) mostly showed a significant increase in the roasted peanuts. The exception was that MGO content was lower at 200 °C than that for 180 °C due to the larger consumption of MGO for the Maillard reaction at higher temperatures. The formation of AGEs and HAs in peanuts also take place rapidly and simultaneously at 180–200 °C. Furthermore, higher roasting temperatures led to the faster formation rates of CML and CEL, and the formation rate of CML was faster than that of CEL, which was related to the higher activation energies for the formation of CEL than that of CML. Four kinds of HAs were detected in roasted peanuts. And Harman and Norharman accounted for about 93% of the total HAs contents in both peanuts after being roasted at 200 °C for 30 min. Moreover, correlation analysis among AGEs, HAs and the active intermediates (i.e., GO and MGO) were conducted. It indicated that CML/CEL showed a linear relationship not only with GO and MGO, but also with HAs in roasted peanuts, while GO and MGO showed a linear relationship not only with CML/CEL, but also with HAs. However, AGEs and HAs contents showed no significant difference between HO−peanut and C−peanut after being roasted, though GO and MGO contents in roasted HO−peanut were mostly more than those in roasted C−peanut. This study will provide a theoretical basis for simultaneously controlling the level of AGEs and HAs in peanut thermal food processing.

## Figures and Tables

**Table 1:** Method validation results of AGEs, HAs, GO and MGO.

Compound	Linear Range (ng/mL)	Calibration Curve Measurement Points	Linear Regression Equation	*R* ^2^	LOD (mg/kg)	LOQ (mg/kg)	Recovery (%)	Precision (RSD,%, *n* = 6)
CML	50–3000	7	y = 0.0097x + 0.1760	0.9999	0.1050	0.3225	104.00 ± 3.08 ^a^	5.62
CEL	25–1000	5	y = 0.0081x + 0.0508	0.9997	0.0875	0.2375	115.00 ± 4.23	5.19
AαC	0.1–20	7	y = 1.47x	0.9974	0.0001	0.0003	86.50 ± 2.03	9.43
DMIP	0.57–20	5	y = 0.69x	0.9972	0.0009	0.0029	91.17 ± 3.12	5.25
Harman	0.1–20	7	y = 1.82x	0.9988	0.0001	0.0002	81.91 ± 2.34	4.18
Norharman	0.1–20	7	y = 2.72x	0.9992	0.0002	0.0005	83.76 ± 1.98	4.10
GO	100–2000	6	Y = 0.0901x − 0.0246	0.9999	0.0160	0.0533	98.88 ± 2.01	3.45
MGO	100–2000	6	Y = 0.0225x − 0.6743	0.9997	0.0180	0.0600	96.24 ± 1.75	4.25

^a^: mean ± SD, *n* = 3.

**Table 2:** AGEs and HAs contents of different peanuts before and after roasting ^g^.

Sample	CML (mg/kg)		CEL (mg/kg)		DMIP (µg/kg)		Harman (µg/kg)		Norharman (µg/kg)	
	Raw	Roasted	Raw	Roasted	Raw	Roasted	Raw	Roasted	Raw	Roasted
1	6.73 ± 0.65 _b_	377.56 ± 11.49 _de_	ND ^h^	83.25 ± 2.83 _e_	ND	4.18 ± 0.04 _d_	ND	3.98 ± 0.18 _e_	ND	6.05 ± 0.21 _e_
2	3.48 ± 0.28 _c_	451.84 ± 15.94 _bc_	ND	105.75 ± 10.25 _abc_	ND	3.70 ± 0.31 _e_	ND	7.60 ± 0.71 _b_	ND	9.60 ± 0.00 _c_
3	6.42 ± 0.23 _b_	302.98 ± 27.75 _f_	ND	83.13 ± 5.48 _e_	ND	6.53 ± 0.39 _c_	ND	5.50 ± 0.21 _d_	ND	8.08 ± 0.39 _d_
4	6.81 ± 0.48 _b_	339.14 ± 19.45 _ef_	ND	81.38 ± 3.71 _e_	ND	6.18 ± 0.32 _c_	ND	5.45 ± 0.57 _d_	ND	6.73 ± 0.39 _e_
5	6.22 ± 0.72 _b_	491.80 ± 53.03 _b_	ND	114.63 ± 5.83 _a_	ND	11.90 ± 0.07 _b_	ND	5.95 ± 0.49 _cd_	ND	7.74 ± 0.46 _d_
6	6.27 ± 0.67 _b_	540.47 ± 27.40 _a_	ND	108.63 ± 3.01 _ab_	ND	12.45 ± 0.21 _a_	ND	8.28 ± 0.53 _b_	ND	11.45 ± 0.42 _b_
7	4.34 ± 0.28 _c_	454.92 ± 24.75 _bc_	ND	88.25 ± 0.09 _de_	ND	3.95 ± 0.21 _de_	ND	3.48 ± 0.39 _e_	ND	4.03 ± 0.39 _f_
8	6.10 ± 0.34 _b_	422.13 ± 14.14 _cd_	ND	98.63 ± 5.48 _bc_	ND	7.48 ± 0.18 _c_	ND	16.20 ± 0.21 _a_	ND	15.23 ± 0.81 _a_
9	8.02 ± 0.94 _a_	381.15 ± 28.28 _de_	ND	95.63 ± 5.83 _cd_	ND	2.85 ± 0.21 _f_	ND	5.80 ± 0.49 _d_	ND	4.83 ± 0.32 _f_
10	ND	428.28 ± 11.59 _c_	ND	84.50 ± 8.13 _e_	ND	2.78 ± 0.11 _f_	ND	6.63 ± 0.46 _c_	ND	6.68 ± 0.04 _e_

^g^: peanut roasting condition: 180 °C, 30 min; the data are shown as mean ± SD, *n* = 3. ^h^: not detected (ND); _a–f_: different letters indicate significant differences (*p* < 0.05).

**Table 3:** HAs contents in C−peanut under different roasting conditions (µg/kg).

Condition		AαC	DMIP	Harman	Norharman	Total
160 °C	10 min	ND	ND	ND	ND	ND
	20 min	ND	ND	1.05 ± 0.07 b	1.88 ± 0.05 b	2.93 ± 0.12 b
	25 min	ND	ND	1.20 ± 0.14 b	2.14 ± 0.19 b	3.34 ± 0.33 b
	30 min	ND	0.82 ± 0.07	2.13 ± 0.11 a	3.93 ± 0.12 a	6.87 ± 0.30 a
180 °C	10 min	ND	0.44 ± 0.00 c	0.65 ± 0.07 d	2.79 ± 0.25 d	4.58 ± 0.32 d
	20 min	ND	1.10 ± 0.08 b	2.35 ± 0.21 c	3.75 ± 0.12 c	7.20 ± 0.25 c
	25 min	ND	2.61 ± 0.00 a	4.35 ± 0.07 b	6.02 ± 0.62 b	12.98 ± 0.69 b
	30 min	ND	3.70 ± 0.31 a	7.60 ± 0.71 a	9.60 ± 0.00 a	20.90 ± 1.01 a
200 °C	10 min	ND	0.47 ± 0.05 d	1.35 ± 0.07 d	1.57 ± 0.00 d	2.69 ± 0.12 d
	20 min	ND	2.69 ± 0.10 c	5.36 ± 0.49 c	6.98 ± 0.25 c	15.01 ± 0.64 c
	25 min	ND	5.00 ± 0.31 b	10.20 ± 0.14 b	19.37 ± 0.49 b	34.57 ± 0.94 b
	30 min	0.80 ± 0.07	8.41 ± 0.62 a	33.95 ± 2.76 a	83.76 ± 7.90 a	126.92 ± 10.11 a

a–d: Different letters indicate significant differences (*p* < 0.05). The data are shown as mean ± SD, *n* = 3. ND-not detected.

**Table 4:** HAs contents in HO−peanut under different roasting conditions (µg/kg).

Condition		AαC	DMIP	Harman	Norharman	Total
160 °C	10 min	ND	ND	ND	ND	ND
	20 min	ND	ND	ND	ND	ND
	25 min	ND	ND	0.60 ± 0.07	0.63 ± 0.04	1.23 ± 0.04
	30 min	ND	ND	0.75 ± 0.07	0.80 ± 0.07	1.55 ± 0.00
180 °C	10 min	ND	ND	ND	ND	ND
	20 min	ND	ND	0.75 ± 0.00 c	0.90 ± 0.07 c	1.65 ± 0.07 c
	25 min	ND	0.95 ± 0.07	2.18 ± 0.18 b	1.65 ± 0.07 b	4.78 ± 0.18 b
	30 min	ND	2.78 ± 0.11	6.63 ± 0.46 a	6.68 ± 0.04 a	16.08 ± 0.53 a
200 °C	10 min	ND	ND	ND	ND	ND
	20 min	ND	1.35 ± 0.07 c	3.23 ± 0.11 c	3.05 ± 0.14 c	7.63 ± 0.00 c
	25 min	ND	3.75 ± 0.35 b	7.95 ± 0.28 b	12.35 ± 0.71 b	24.05 ± 1.34 b
	30 min	0.55 ± 0.00	4.78 ± 0.25 a	20.63 ± 1.17 a	45.95 ± 1.56 a	71.90 ± 2.97 a

a–c: Different letters indicate significant differences (*p* < 0.05). The data are shown as mean ± SD, *n* = 3. ND-not detected.

**Table 5:** Mass spectrometry parameters of target compounds and internal standards.

Analytes	Precursor Ion (*m*/*z*)	Production Ion (*m*/*z*)	CE (v)	DP (v)	Dwell Time (ms)
CML	205.0	130.0 ^d^	56	17	50
		84.0	56	25	50
CEL	219.0	130.0	67	18	50
		84.0	66	29	50
CML-*d*_4_	209.0	134.0	50	19	50
		88.0	70	29	50
CEL-*d*_4_	223.0	134.0	70	19	50
		88.0	70	29	50
AαC	184.0	167.2	108	32	30
		140.0	108	32	30
DMIP	162.9	147.3	90	45	30
		105.0	90	45	30
4,7,8-TriMeIQx	242.0	227.1	120	40	30
		145.0	120	50	30
Harman	183.0	115.0	120	50	30
		168.3	120	40	30
Norharman	169.2	115.0	100	45	30
		142.0	100	40	30

d: Quantitative ion.

## Data Availability

Not applicable.

## Supplementary Materials

The following supporting information can be downloaded at https://www.mdpi.com/article/10.3390/molecules28207012/s1. Table S1: The contents of reducing sugars in peanuts after the roasting process; Figure S1: The contents of reducing sugars in peanuts after the roasting process.

## Sample Availability

Samples are available from Wei Liu on valid request.
