# Supplementary material for: Comprehensive Analyses of Advanced Glycation end Products and Heterocyclic Amines in Peanuts during the Roasting Process
Source: Molecules. 2023 Oct 10;28(20):7012. doi: 10.3390/molecules28207012 (PMC10608810; doi:10.3390/molecules28207012)
Supplement: Supplementary file 1 [file molecules-28-07012-s001.zip › molecules-2585585-supplementary.pdf]

Comprehensive Analyses of Advanced Glycation end Products and Heterocyclic Amines in Peanuts during the Roasting Process

Jingjing Yu [1,*], Xiaohui Yu [2], Lili Shi [2] and Wei Liu [2,*]

Table S1. The contents of reducing sugars in peanuts after roasting process (g/kg).

Peanut	Roasting time	160°C	180°C	200°C
C-peanut	0min	2.23±0.01[a]	2.23±0.01[a]	2.23±0.01[a]
	10min	2.17±0.04[ab]	2.01±0.15[bc]	1.84±0.10[b]
	15min	2.10±0.07[b]	2.15±0.06[ab]	1.87±0.02[b]
	20min	2.12±0.07[ab]	2.11±0.07[ab]	1.84±0.01[b]
	25min	2.09±0.06[b]	1.86±0.02[cd]	2.14±0.03[a]
	30min	2.20±0.01[ab]	1.77±0.03[d]	2.28±0.11[a]
HO-peanut	0min	2.44±0.10[ab]	2.44±0.10[a]	2.44±0.10[c]
	10min	2.24±0.04[b]	2.32±0.04[b]	2.45±0.05[c]
	15min	2.37±0.11[ab]	2.37±0.07[ab]	2.32±0.06[cd]
	20min	2.46±0.13[a]	2.29±0.03[bc]	2.25±0.09[d]
	25min	2.41±0.06[ab]	2.20±0.03[bc]	2.74±0.02[b]
	30min	2.44±0.01[ab]	2.35±0.11[ab]	3.38±0.08[a]

a–d: Different letters indicate significant differences ($p < 0.05$). The data are shown as mean ± SD, n = 3.

		160°C	180°C	200°C
C-peanut	0min	2.23	2.23	2.23
	10min	2.17	2.01	1.84
	15min	2.1	2.15	1.87
	20min	2.12	2.11	1.84
	25min	2.09	1.86	2.14
	30min	2.2	1.77	2.28
HO-peanut	0min	2.44	2.44	2.44
	10min	2.24	2.32	2.45
	15min	2.37	2.37	2.32
	20min	2.46	2.29	2.25
	25min	2.41	2.2	2.74
	30min	2.44	2.35	3.38

Figure S1. The contents of reducing sugars in peanuts after roasting process (g/kg)
